# Statutory Retirement in China: A Thematic Analysis of Weibo Trending Posts in 2024

**DOI:** 10.1093/geroni/igaf122.2763

**Published:** 2025-12-31

**Authors:** Jinyi Xiao, Sonja Amadae

**Affiliations:** University of Helsinki, Helsinki, Uusimaa, Finland; University of Helsinki, Helsinki, Uusimaa, Finland

## Abstract

China recently officially announced a gradual increase in the statutory retirement age for male from 60 to 63 years, and for female from 50 or 55 to 55 and 58 respectively. And the extension of contribution years based on a voluntary and flexible principle. We explored and analyzed the online public responses and discussions of increasing retirement age policy on Chinese social media (Weibo) and also examined how the themes evolved over different stages. This research retrieved 87 topics and 1,115 trending posts from Weibo across two critical policy stages (official announcement and detailed information) from July 22 to September 22, 2024. Inductive thematic analysis was employed to analyze the post content and categorize it into different themes. A frequency approach was used as supplementary to demonstrate and analyze the popularity of identified themes. Seven themes and twenty-four subthemes were identified. The most prominent theme was “subjective well-being concerns, “ which was also the dominant theme in the second stage. “Public Health Concerns” and “Social Inequality” emerged as the second and third dominant themes overall, and were the most prominent themes in the first stage. Public discourse on Weibo regarding retirement age increases reveals this policy is widely perceived as unfriendly, compromising individual interests and exacerbating structural inequalities. The public views it as inherently detrimental to older workers, intensifying rather than addressing existing social disparities.

